# Compact folded metasurface spectrometer

**DOI:** 10.1038/s41467-018-06495-5

**Published:** 2018-10-10

**Authors:** MohammadSadegh Faraji-Dana, Ehsan Arbabi, Amir Arbabi, Seyedeh Mahsa Kamali, Hyounghan Kwon, Andrei Faraon

**Affiliations:** 1 T. J. Watson Laboratory of Applied Physics and Kavli Nanoscience Institute, California Institute of Technology, 1200 East California Boulevard, Pasadena, CA 91125 USA; 2 Department of Electrical and Computer Engineering, University of Massachusetts Amherst, 151 Holdsworth Way, Amherst, MA 01003 USA

## Abstract

An optical design space that can highly benefit from the recent developments in metasurfaces is the folded optics architecture where light is confined between reflective surfaces, and the wavefront is controlled at the reflective interfaces. In this manuscript, we introduce the concept of folded metasurface optics by demonstrating a compact spectrometer made from a 1-mm-thick glass slab with a volume of 7 cubic millimeters. The spectrometer has a resolution of ~1.2 nm, resolving more than 80 spectral points from 760 to 860 nm. The device is composed of three reflective dielectric metasurfaces, all fabricated in a single lithographic step on one side of a substrate, which simultaneously acts as the propagation space for light. The folded metasystem design can be applied to many optical systems, such as optical signal processors, interferometers, hyperspectral imagers, and computational optical systems, significantly reducing their sizes and increasing their mechanical robustness and potential for integration.

## Abstract (web summary)

Here, the authors introduce the folded metasurface optics architecture by demonstrating a compact high-resolution optical spectrometer made from a 1-mm-thick glass slab. The spectrometer has a resolution of 1.2 nm, resolving more than 80 spectral points in a 100-nm bandwidth centered at 810 nm.

## Introduction

**Fig. 1:**
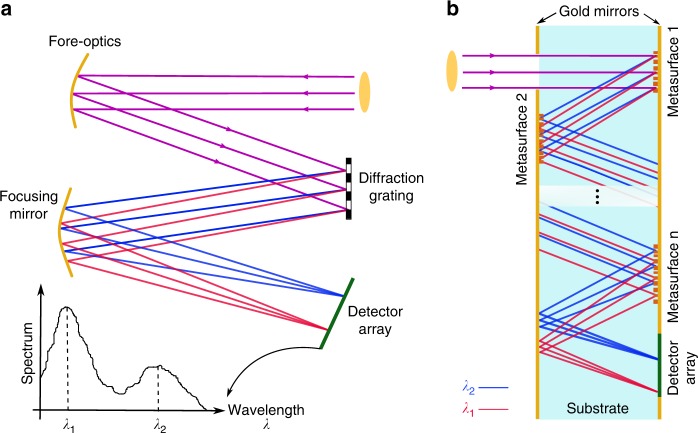
Schematics of a conventional and a folded metasurface spectrometer. **a** Schematic illustration of a typical diffractive spectrometer. The main components are comprised of the fore-optics section, diffraction grating, focusing lenses, and detector array. **b** The proposed scheme for a folded compact spectrometer. All the dispersive and focusing optics can be implemented as reflective metasurfaces on the two sides of a single transparent substrate. Mirrors on both sides confine and direct light to propagate inside the substrate, and the detector can be placed directly at the output aperture of the device. If required, transmissive metasurfaces can also be added to the input and output apertures to perform optical functions. Although the schematic here includes metasurfaces on both sides to show the general case, the actual devices demonstrated here are designed to have metasurfaces only on one side to simplify their fabrication

Optical spectrometry is a key technique in various areas of science and technology with a wide range of applications^1,2^. This has resulted in a large demand for spectrometers and/or spectrum analyzers with different properties (e.g., operation bandwidth, resolution, size, etc.) required for different applications^3–5^. Conventional optical spectrometers are composed of a dispersive element, such as a prism or a diffraction gating, that deflects different wavelengths of light by different angles, followed by focusing elements that focus light incoming from different angles to different points (or lines). As schematically shown in Fig. 1a, the intensity at different wavelengths can then be measured using an array of detectors. Diffraction gratings have typically larger dispersive powers than transparent materials, and therefore diffractive spectrometers generally have better resolutions^1^. The combination of several free-space optical elements (the grating, focusing mirrors, etc.) and the free-space propagation volume result in bulky spectrometers. In recent years, there has been an increased interest in high-performance compact spectrometers that can be easily integrated into consumer electronics for various medical and technological applications such as medical diagnosis, material characterization, quality control, etc.^6,7^. As a result, various schemes and structures have been investigated for realization of such spectrometers^7–16^. One class of miniaturized spectrometers integrate a series of band-pass filters with different center wavelengths on an array of photodetectors^8,17^. Although these devices are compact and compatible with standard microfabrication techniques, they have resolutions limited by achievable filter quality factors, and low sensitivities caused by the filtering operation that rejects a large portion of the input power. Spectrometers based on planar on-chip integrated photonics provide another solution with high spectral resolution^7,9–13^. However, the loss associated with on-chip coupling of the input light and the reduced throughput because of the single-mode operation^18^ are still major challenges for widespread adoption in many applications.

Another type of compact spectrometers are conceptually similar to the conventional table-top spectrometers, however, they use micro-optical elements to reduce size and mass^14,15^. Due to the inferior quality and limited control achievable by micro-optical elements as well as the shorter optical path lengths, these devices usually have lower spectral resolutions. Higher resolution has been achieved by using aberration-correcting planar gratings^16^, however an external spherical mirror makes the device bulky.

Dielectric metasurfaces, a new category of diffractive optical elements with enhanced functionalities, have attracted a great deal of interest in recent years^19–22^. Overcoming many of the material and fundamental limitations of plasmonic metasurfaces^23^, dielectric metasurfaces have proven capable of implementing several conventional^19,24–34^ and new optical devices^35–39^ with high efficiencies. They enable control of phase with subwavelength resolution and high gradients and simultaneous control of phase and polarization^35^. A key feature of metasurfaces is their compatibility with micro and nano-fabrication techniques, which allows for integration of multiple metasurfaces for realizing complex optical metasystems^24,40^. Such metasystems allow for significantly improving optical properties of metasurfaces through aberration correction (such as lenses with diffraction limited operation over wide field of view^24^), or functionalities fundamentally unachievable with local single-layer metasurfaces such as retroreflection^40^.

## Results

### Concept and design

Taking a different approach to device integration, here we introduce folded optical metasystems where multiple metasurfaces are integrated on a single substrate that is also playing the role of propagation space for light [Fig. 1b]. Using this platform, we experimentally demonstrate a compact folded optics device for spectroscopy with a 1-mm thickness (**~**7-mm^3^ volume) that provides a **~**1.2-nm resolution over a 100-nm bandwidth (more than 80 points over a **~**12% bandwidth) in the near-infrared. As schematically shown in Fig. 1b, multiple reflective metasurfaces can be designed and fabricated on the same transparent substrate to disperse and focus light to different points on a plane parallel to the substrate. To the best of our knowledge, this is the first demonstration of an optical metasystem comprising more than two metasurfaces that implements a sophisticated optical functionality like spectrometry. Furthermore, the presented configuration can allow for the integration of the detector array on top of the folded spectrometer, resulting in a compact monolithic device. We should note here that it was recently demonstrated that an off-axis metasurface lens (i.e., a lens with an integrated blazed grating phase profile^41,42^) can disperse and focus different wavelengths to different points. However, there are fundamental and practical limitations for such elements that significantly limits their application as a spectrometer (which is the reason why other types of diffractive optical elements, such as holographic optical elements and kinoforms, that can essentially perform the same function have not been used for this application before). Fundamentally, the chromatic dispersion^43–47^ and angular response correlation of diffractive optical elements and metasurfaces^38,48^ limit the bandwidth and angular dispersion range where the device can provide tight aberration-free focusing. This in turn limits the achievable resolution and bandwidth of the device. Moreover, the chromatic dispersion results in a focal plane almost perpendicular to the metasurface, which will then require the photodetector array to be placed almost normal to the metasurface plane^41,42,49^. In addition to the distance for the propagation of dispersed light, this normal placement undermines the compactness of the device.

**Fig. 2:**
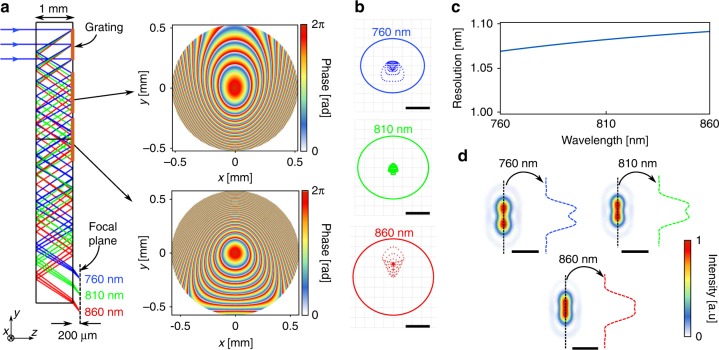
Ray-optics design and simulation results of the folded spectrometer. **a** Ray-tracing simulation results of the folded spectrometer, shown at three wavelengths in the center and two ends of the band. The system consists of a blazed grating that disperses light to different angles, followed by two metasurfaces optimized to focus light for various angles (corresponding to different input wavelengths). The grating has a period of 1 μm, and the optimized phase profiles for the two metasurfaces are shown on the right. **b** Simulated spot diagrams for three wavelengths: center and the two ends of the band. The scale bars are 5 μm. **c** Spectral resolution of the spectrometer, which is calculated from simulated Airy disk radii and the lateral displacement of the focus with wavelength. **d** Simulated intensity distribution for two wavelengths separated by 1.1 nm around three different center wavelengths of 760, 810, and 860 nm. The intensity distributions show that wavelengths separated by 1.1 nm are theoretically resolvable. The scale bars are 20 μm

Figure 2a shows the ray-tracing simulations of the designed spectrometer. The device consists of three metasurfaces, all patterned on one side of a 1-mm-thick fused silica substrate. The first metasurface is a periodic blazed grating with a period of 1 μm that disperses different wavelengths of a collimated input light to different angles, centered around 33.9° at 810 nm. The second and third metasurfaces focus light coming from different angles (corresponding to various input wavelengths) to different points on the focal plane. We have recently demonstrated a metasurface doublet capable of correcting monochromatic aberrations to achieve near-diffraction-limited focusing over a wide field of view^24^. The second and third metasurfaces here essentially work similar to the mentioned doublet, with the difference of working off-axis and being designed in a folded configuration, such that the focal plane for our desired bandwidth is parallel to the substrate. To simplify the device characterization, the focal plane was designed to be located 200 μm outside the substrate. The asymmetric design of the focusing metasurfaces in an off-axis doublet configuration, allows for the focal plane to be parallel to the substrate. This makes the integration of the spectrometer and the detector array much simpler, results in a more compact and mechanically robust device, and allows for direct integration into consumer electronic products like smartphones. The optimized phase profiles for the two surfaces are shown in Fig. 2a, right (see Supplementary Table 1 for the analytical expression of the phase). Simulated spot diagrams of the spectrometer are plotted in Fig. 2b for three wavelengths at the center and the two ends of the bandwidth showing negligible geometric aberrations. The spot diagrams are plotted only at three wavelengths, but the small effect of optical aberrations was confirmed for all wavelengths in the 760–860 nm bandwidth. As a result, the spectral resolution of the device can be calculated using the diffraction limited Airy radius and the lateral displacement of the focus by changing the wavelength. The calculated resolution is plotted in Fig. 2c, showing a theoretical value of better than 1.1 nm across the band. Point spread functions (PSFs) calculated for input beams containing two wavelengths 1.1 nm apart, and centered at 760, 810, and 860 nm are plotted in Fig. 2d, showing two resolvable peaks.

**Fig. 3:**
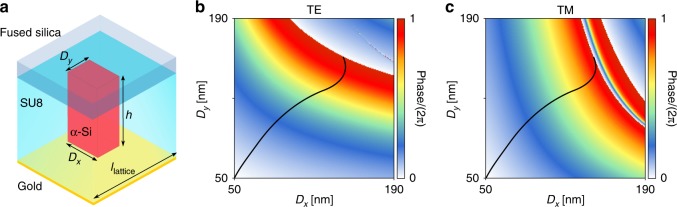
Metasurface structure and design graphs. **a** Schematics of the reflective rectangular meta-atom. The meta-atom consists of *α*-Si nano-posts on a fused silica substrate, capped by a layer of SU-8 polymer and backed by a gold mirror. The nano-post is 395 nm tall and the lattice constant is 250 nm for the blazed grating and 246 nm for the focusing metasurfaces. **b**, **c** Simulated reflection phase plotted for TE and TM polarizations. The black curve highlights the path through the *D*_x_ − *D*_y_ plane that results in equal phases for the two polarizations. Nano-posts on this path were used to design the two focusing metasurface elements to make them insensitive to the input polarization

To implement the reflective metasurfaces, we used a structure similar to the reflective elements in ref. ^40^. Each of the meta-atoms, shown schematically in Fig. 3a, consists of an *α*-Si nano-post with a rectangular cross-section, capped by a 2-μm-thick SU-8 layer and backed by a gold mirror. The post height and lattice constant were chosen to be 395 and 246 nm, respectively, to achieve full 2*π* phase coverage while minimizing variation of the reflection phase derivative across the band (Supplementary Fig. 1). Minimizing the phase derivative variation will mitigate the reduction of device efficiency over the bandwidth of interest^47^ by decreasing the wavelength dependence of the phase profiles (Supplementary Fig. 2). In addition, since the two focusing metasurfaces are working under an oblique illumination (*θ* ~ 33.9°), the nano-posts were chosen to have a rectangular cross-section to minimize the difference in reflection amplitude and phase for the transverse electric (TE) and transverse magnetic (TM) polarizations (for the oblique incident angle of 33.9° at 810 nm). Reflection coefficients are found through simulating a uniform array of nano-posts under oblique illumination (*θ* ~ 33.9°) with TE and TM polarized light. The simulated reflection phase as a function of the nano-posts side lengths are shown in Fig. 3b, c for TE and TM polarizations. The black curve highlights the path through the *D*_x_-*D*_y_ plane along which the reflection phases for the TE and TM polarizations are almost equal. In addition, as shown in Supplementary Fig. 2 having almost the same reflection phases for the TE and TM polarizations holds true for the whole desired 760 nm–860 nm bandwidth. The nano-post dimensions calculated from this path were used to implement the two focusing metasurfaces.

The blazed grating has a periodic phase profile (with a period of 1 μm) that deflects normally incident light to a large-angle inside the substrate. With a proper choice of the lattice constant (250 nm, in our case), its structure can also be periodic. This different structure and operation require a different design approach. The periodicity of the grating allows for its efficient full-wave simulation, which can be used to optimize its operation over the bandwidth of interest. A starting point for the optimization was chosen using the recently developed high-NA lens design method^50^, and the structure was then optimized using the particle swarm optimization algorithm to simultaneously maximize deflection efficiency at a few wavelengths in the band for both polarizations (see Supplementary Note 1 and Supplementary Fig. 3 for details).

### Device fabrication

**Fig. 4:**
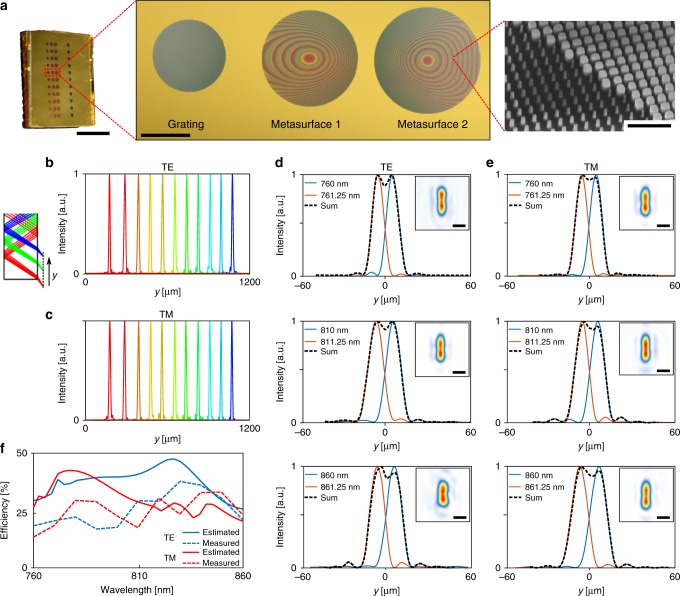
Experimental characterization results. **a** An optical microscope image of the fabricated device and metasurfaces before deposition of the second gold layer. Inset shows a scanning electron micrograph of a portion of one of the two focusing metasurfaces (scale bars from left to right: 10 mm, 500 μm, and 1 μm). **b**, **c** One-dimensional focal spot profiles measured for several wavelengths in the bandwidth along the *y*-direction (as indicated in the inset) for TE and TM polarizations. The wavelengths start at 760 nm (blue curve) and increase at 10-nm steps up to 860 nm (red curve). **d**, **e** Measured intensity distributions for two input wavelengths that are 1.25 nm apart for TE and TM polarizations. The measurements were carried out at the center and at  the two ends of the wavelength range for both polarizations. The insets show the corresponding 2-dimensional intensity profiles, demonstrating two resolvable peaks (scale bars: 10 μm). **f** Calculated and measured absolute focusing efficiencies of the spectrometer for TE and TM polarizations. Both polarizations have average measured efficiencies of ~25%

The device was fabricated using conventional micro- and nano-fabrication techniques (Supplementary Fig. 4). First, a 395-nm-thick layer of *α*-Si was deposited on a 1-mm-thick fused silica substrate. All metasurfaces were then patterned using electron beam lithography in a single step, followed by a pattern inversion through the lift-off and dry etching processes. The metasurfaces were capped by a ~2-μm-thick SU-8 layer, and a 100-nm-thick gold layer was deposited as the reflector. A second reflective gold layer was deposited on the second side of the substrate. Both the input and output apertures (with diameters of 790 and 978 μm, respectively) were defined using photolithography and lift-off. An optical microscope image of the three metasurfaces, along with a scanning electron micrograph of a part of the fabricated device are shown in Fig. 4a.

### Experimental and characterization results

To experimentally characterize the spectrometer, a normally incident collimated beam from a tunable continuous wave laser was shined on the input aperture of the device. A custom-built microscope was used to image the focal plane of the spectrometer, ~200 μm outside its output aperture (see Supplementary Note 3 and Supplementary Fig. 5 for details of the measurement setup). The input wavelength was tuned from 760 to 860 nm in steps of 10 nm, and the resulting intensity distributions were imaged using the microscope. The resulting one-dimensional intensity profiles are plotted in Fig. 4b, c for TE and TM polarizations. The intensity profiles were measured over the whole 1.2-mm length of the *y*-direction in the focal plane (as shown in Fig. 4, inset) at each wavelength. The background intensity is beyond visibility in the linear scale profiles plotted here for all wavelengths (see Supplementary Figs. 6, 7 for two-dimensional and logarithmic-scale plots of the intensity distribution, respectively). Figure 4d, e show the measured intensity profiles for three sets of close wavelengths, separated by 1.25 nm. The insets show the corresponding two-dimensional intensity distribution profiles. For all three wavelengths, and for both polarizations the two peaks are resolvable. The experimentally obtained spectral resolution is plotted in Supplementary Fig. 8 vs. the wavelength. The average resolution for both polarizations is ~1.2 nm, which is slightly worse than the theoretically predicted value (~1.1 nm). We attribute the difference mostly to practical imperfections such as the substrate having an actual thickness different from the design value and thickness variation. In addition, the metasurface phases are slightly different from the designed profiles due to fabrication imperfections. The angular sensitivity/tolerance of the device was also measured with respect to polar and azimuthal angle deviations from 0 incidence angle, in the *x*-*z* and *y*-*z* planes, using the setup shown in Supplementary Fig. 9c. In the *y*-*z* plane the maximum tilt angle to maintain the same 1.25 nm resolution is ±0.15°, while in the *x*-*z* plane the device has a ±1° degree acceptance angle. The measurement results in Supplementary Fig. 9 match well with the predictions from ray-tracing simulations.

The measured and calculated focusing efficiencies are plotted in Fig. 4f. The focusing efficiency, defined as the power passing through a ~30- μm diameter pinhole around the focus divided by the total power hitting the input aperture, was measured using the setup shown in Supplementary Fig. 5. For both polarizations, the average measured efficiency is about 25%. As seen from the measured efficiency curves, the optimization of the blazed grating efficiency vs. wavelength and the choice of the design parameters to minimize variations in the phase-dispersion for the doublet metasurface lens, have resulted in a smooth measured efficiency. An estimate for the expected efficiency (shown as simulated efficiency in Fig. 4f) is calculated by multiplying the deflection efficiency of the grating, the efficiency of seven reflections off the gold mirrors, the input and output aperture transmission efficiencies, and the average reflectivities of the uniform nano-post arrays (as an estimate for the two focusing metasurface efficiencies). It is worth noting that considering only the reflection losses at the interfaces (nine reflective ones, and two transmissive ones) reduces the efficiency to about 48%, showing a close to 50% efficiency for the three metasurfaces combined. We attribute the remaining difference between the measured and estimated values to fabrication imperfections (e.g., higher loss for the actual gold mirrors, and imperfect fabrication of the metasurfaces), the lower efficiency of the metasurfaces compared to the average reflectivity of uniform arrays, and to the minor difference from the designed value of the metasurface phase profiles at wavelengths other than the center frequency.

### Spectrum measurement

**Fig. 5:**
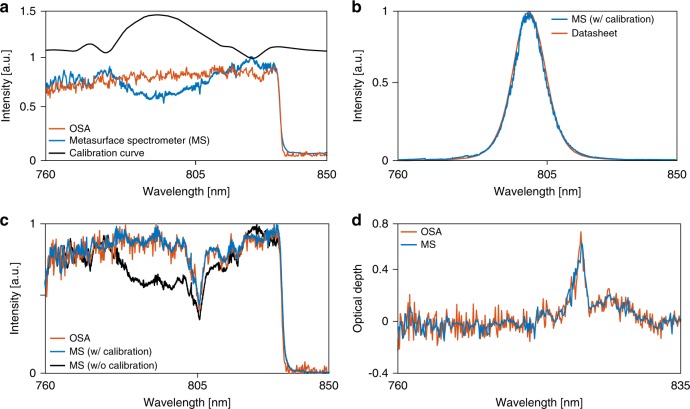
Sample spectrum measurement results. **a** Spectrum of a wideband source (a super-continuum laser with an 840-nm short-pass filter) measured by a commercial OSA and the metasurface spectrometer (MS). This measurement was used to extract the calibration curve. **b** Spectrum of a 10-nm band-pass filter centered at 800 nm measured by the MS, compared to the spectrum acquired from the filter datasheet. **c** Transmission spectrum of a Nd:YVO_4_ crystal sample measured both with a commercial OSA and the MS. **d** The optical depth of the sample extracted from the spectrum measurements both with the OSA and the MS

To demonstrate that the metasurface spectrometer actually has the ability to measure dense optical spectra, we use it to measure the transmission spectra of two different samples. First, we measured the spectrum of a wideband source (a super-continuum laser source, filtered with an 840-nm short-pass filter), both with the metasurface spectrometer (MS) and a commercial optical spectrum analyzer (OSA). By dividing the spectra measured by the two devices, we extract the required calibration curve that accounts for the variation of the metasurface spectrometer as well as the non-uniformities in the responsivity of the optical setup used to image the focal plane (i.e., the objective lens and the camera, as well as the optical fiber used to couple the signal to the OSA). The measured spectra and the extracted calibration curve are plotted in Fig. 5a. Next, we used this calibration curve to measure the transmission spectrum of a band-pass filter with a nominal 10-nm full width at half maximum bandwidth and centered at 800 nm. The measured spectrum along with the transmission spectrum obtained from the filter datasheet are plotted in Fig. 5b, showing a good agreement. Finally, we used the metasurface spectrometer to measure the optical depth of a Nd:YVO_4_ crystal sample. The spectrum measured with the metasurface spectrometer (after calibration) is compared with the transmission spectrum of the same sample measured with the OSA in Fig. 5c. Dividing the spectrum without and with the sample, we have extracted the optical depth of the sample, which is plotted in Fig. 5d. A good agreement is observed between the two measurement results. It is worth noting that the Nd:YVO_4_ crystal sample was cut though the *z*-plane, resulting in an equal absorption spectrum for the two polarizations. Therefore, we can assume that all spectral measurements were done with the same state of input polarization. This justifies the use of only one calibration curve for all the measurements.

## Discussion

The measured efficiency of the spectrometer demonstrated here is about 25%. This value can be significantly increased to about 70% by using mirrors with higher reflectivity (e.g., distributed Bragg reflectors (DBRs) or high-contrast grating mirrors^51,52^), and anti-reflection coatings on the input and output apertures. In addition, more advanced optimization techniques^53^ could be exploited to optimize the diffraction grating to achieve high efficiency and polarization insensitivity. Implementing these changes and optimizing the fabrication process, one can expect to achieve efficiencies exceeding 70% for the spectrometer.

The metasurface spectrometers are fabricated in a batch process, and, therefore, many of them can be fabricated on the same chip, even covering multiple operation bandwidths. This can drastically reduce the price of these devices, allowing for their integration into various types of systems for different applications. In addition, the demonstrated structure is compatible with many of the techniques developed for the design of multi-wavelength metasurfaces^46,54^, and, therefore, one might be able to combine different optical bandwidths into the same device (e.g., using a grating that deflects to the right at one bandwidth, and to the left at the other), resulting in compact devices with enhanced functionalities.

The optical throughput (etendue) is a fundamental property of any optical system, setting an upper limit on the ability of the system to accept light from spatially incoherent sources. It can be estimated as the product of the physical aperture size and the acceptance solid angle of the system. Furthermore, the total etendue of a system is limited by the element with the lowest etendue. To calculate the throughput of the metasurface spectrometer, we have performed simulations and measurements to characterize its acceptance angle. According to the measurement, results in Supplementary Fig. 9 the acceptance angle of the system is about 2 degrees in the horizontal direction, and 0.3 degrees in the vertical direction. Given this and the input aperture dimensions, the optical throughput of our device is calculated to be ~90 Sr(μm)^2^. For comparison, the etendue of optical systems operating around 1 μm that utilize single-mode input channels (i.e., most optical spectrometers based on integrated optics platforms) is around ~1 Sr(μm)^2^. Furthermore, the demonstrated spectrometer is optimized for maximum sensitivity and not throughput. To show that the achieved throughput here does not denote an upper limit for the etendue of a folded metasurface spectrometer with similar characteristics (i.e., resolution, bandwidth, etc.), we have designed a second device with a throughput of ~13,000 Sr(μm)^2^ (see Supplementary Note 5 and Supplementary Fig. 10 for design details and simulation results). Table 1 provides a comparison of the optical throughput of several compact spectrometers from recent literature. According to Table 1, the spectrometers designed using the folded metasurface platform can collect 2 to 4 orders of magnitude more light compared to on-chip spectrometers that are based on single/few-mode input waveguides, resulting in a much higher sensitivity.

**Table 1:** Comparison of different spectrometers in terms of throughput (Etendue) and their dimensions

Spectrometer	Etendue [Sr(μm)^2^]	Size (dimensions)
[11]	<0.5	50 μm × 100 μm × thickness
[55]	∼0.8	16 mm × 7 mm × 15 μm
This work	∼90	1 mm × 1 mm × 7 mm
[15]	∼8250	20.1 mm × 12.5 mm × 10.1 mm
Supplementary Fig. 10	∼13,000	2 mm × 2.5 mm × 8 mm

The development of thin and compact optical elements and systems has been a key promise of optical metasurfaces. Although many optical devices have been developed in thin and compact form factors using metasurfaces, significantly reducing the volume of optical systems using metasurfaces has not been previously demonstrated due to the requirement of the free-space propagation in many systems (e.g., imaging systems, spectrometers, etc.). The folded metasystem configuration introduced here can significantly reduce the size of many of these optical systems using the substrate as the propagation space for light. Based on this configuration, we demonstrated a 1-mm-thick spectrometer with a 7-mm^3^ volume, reduced by a factor of ten compared to the same system implemented in an unfolded scheme (twenty times reduction, if the same system was designed in air). The spectrometer has a resolution of ~1.2 nm over a 100-nm bandwidth (>12%) in the near-infrared. Using this design, multiple spectrometers can be fabricated on the same chip and in the same process, significantly reducing the costs and enabling integration of spectrometers covering multiple optical bands into consumer electronics. Moreover, by improving the angular response of the current device one can design a compact hyperspectral imager capable of simultaneous one-dimensional imaging and spectroscopy. In a broader sense, we expect that the proposed platform will also be used for on-chip interferometers, imaging systems, and other devices performing complex transformations of the field.

## Electronic supplementary material

Supplementary Information

Peer Review File

## Data Availability

The data that support the findings of this study are available from the corresponding author upon request.
