# Supplementary material for: Compact folded metasurface spectrometer
Source: Nat Commun. 2018 Oct 10;9:4196. doi: 10.1038/s41467-018-06495-5 (PMC6180047; doi:10.1038/s41467-018-06495-5)
Supplement: Supplementary file 1 — Supplementary Information [file 41467_2018_6495_MOESM1_ESM.pdf]

Compact Folded Metasurface Spectrometer: Supplementary Material

MohammadSadegh Faraji-Dana, Ehsan Arbabi, Amir Arbabi,
Seyedeh Mahsa Kamali, Hyounghan Kwon, and Andrei Faraon

SUPPLEMENTARY NOTE 1: SIMULATION AND DESIGN

Ray tracing simulations of the spectrometer were performed using Zemax OpticStudio. In the simulations, metasurfaces were assumed to be phase-only diffractive surfaces. The grating was modeled as a blazed grating with a linear phase along the direction of dispersion (y), and independent of the other direction (x). The phase was chosen to correspond to a period of 1 μm, resulting in deflection angles of 31.6° and 36.35° at 760 nm and 860 nm, respectively. The angles were chosen such that the focused light could be captured by an objective with a numerical aperture of 0.95, while maximizing the dispersive power. The second and third surfaces were modeled as a summation of Cartesian coordinate polynomials (Binary 1), $\Sigma_{n,m} a_{m,n} x^m y^n$, and cylindrical coordinate radially symmetric polynomials (Binary 2) $\Sigma_i b_{2i} \rho^{2i}$. The coefficients were optimized to reduce geometric aberrations by minimizing the root mean square geometric spot radii for several input wavelengths covering the bandwidth. The optimized coefficients are given in Supplementary Table **1**. As shown in to Fig. **2**b, all focal spots are optimized and are within the airy disks. This indicates that the designed spectrometer has small geometrical aberrations. The diffraction-limited resolution curve obtained is shown in Fig. **2**c. The simulations and optimizations were first performed in an unfolded configuration for simplicity. There were several constraints in finding the sizes for input and output apertures. Two opposing factors existed in determination of the 790-μm input aperture diameter. On one hand, a larger input aperture results in a higher throughput and more captured light as well as a higher numerical aperture and potentially better resolution. On the other hand, the aperture size for the folded platform cannot be arbitrarily large because different metasurfaces should not overlap. Thus, the 790-μm aperture diameter was chosen in the ray-tracing simulations as the largest size for which metasurface overlap can be avoided and diffraction-limited focusing can be achieved. The output aperture spatially filters the out of band wavelengths while passing through the bandwidth of interest. Therefore, its size was chosen as the smallest possible aperture that allows for all wavelengths of interest to pass through. Using the ray-tracing simulations, this optimum size was found to be 978 μm.

The rigorous coupled wave analysis (RCWA) technique [1] was used to obtain reflection phases of the nano-posts. For each specific set of dimensions, a uniform array of the α-Si nano-posts was illuminated with a plane wave at the wavelength of 810 nm under an illumination angle of 33.9° and the reflected amplitudes and phases were extracted for each polarization. To choose the height of the nano-posts, we performed these simulations for nano-posts with square cross-

sections and different heights and side lengths [Supplementary Figure **1**]. The height was then chosen to minimize the variation of the derivative of the phase with respect to wavelength for different side lengths, while providing a full 2π phase coverage and high reflectivity. Considering the results of Supplementary Figure **1**b and Supplementary Figure **1**d, we chose the thickness to be 395 nm. Although this height is slightly less than λ/2, it is large enough to provide a full 2π phase coverage as the device operates in reflection mode. The lattice constant was chosen to be 246 nm in order to satisfy the sub-wavelength condition and avoid higher order diffractions, which require $l_c < \lambda/n(1 + \sin(\theta_{\text{max}}))$, where l_c is the lattice constant, n is the refractive index of the substrate, and θ_{max} is the maximum deflection angle [2]. We chose $\sin(\theta_{\text{max}}) = 1/n$, since light traveling at larger angles will undergo total internal reflection at the output aperture. To make the two focusing metasurfaces polarization-insensitive, reflection phase and amplitudes were obtained for nano-posts with rectangular cross section under oblique illumination with both TE and TM polarizations [Fig. **3**]. The design curves were then generated by determining a path in the D_{x}-D_{y} plane along which TE and TM reflection phases are almost equal.

For designing the blazed diffraction grating, we chose to use the same α-Si thickness of 395 nm (for ease of fabrication). The lattice constant was set to be 250 nm, such that a grating period contains four nano-posts, and the structure becomes fully periodic. This allows for using periodic boundary conditions in the full-wave simulations of the structure, reducing the simulation domain size significantly. The initial values of the post widths were chosen using a recently developed high-NA metasurface design approach [3]. The simulation results for nano-post-width vs reflection-phase and the initial post widths are plotted in Supplementary Figure **3**a. These values were then fed to a particle swarm optimization algorithm (using an RCWA forward solver) as a starting point. The algorithm optimizes the deflection efficiency of the grating for both polarizations at 11 wavelengths spanning the bandwidth of interest. The optimization parameters are the side lengths of the rectangular nano-posts, while their thickness and spacing is fixed. Deflection efficiencies of the initial and optimized gratings are plotted in Supplementary Figure **3**b. The corresponding nano-post widths for both gratings are given in Supplementary Table **2**.

SUPPLEMENTARY NOTE 2: SAMPLE FABRICATION

A summary of the key steps of the fabrication process is shown in Supplementary Figure **4**. A 395-nm-thick layer of α-Si was deposited on one side of a 1-mm-thick fused silica substrate

through a plasma enhanced chemical vapor deposition process at 200°C. The metasurface pattern was then generated in a $\sim$300-nm-thick layer of ZEP-520A positive electron resist (spun for 1 minute at 5000 rpm) using an EBPG5200 electron beam lithography system. After development of the resist in a developer (ZED-N50, Zeon Chemicals), a $\sim$70-nm-thick alumina layer was evaporated on the sample in an electron beam evaporator. After lift-off, this layer was used as a hard mask for dry etching the α-Si layer in a mixture of SF_6 and C_4F_8 plasma. The alumina layer was then removed in a 1:1 solution of H_2O_2 and NH_4OH. A $\sim$2-μm-thick layer of SU-8 2002 polymer was spin-coated, hard-baked and cured on the sample to protect the metasurfaces. The output aperture (which is on the same side as the metasurfaces) was defined using photolithography (AZ-5214E positive resist, MicroChemicals) and lift-off. A $\sim$100-nm-thick gold layer was deposited as the reflective surface. To protect the gold reflector, a second layer of SU-8 2002 was used. To define the input aperture, a $\sim$2-μm-thick layer of SU-8 2002 polymer was spin-coated and cured on the second side of the wafer to improve adhesion with gold. The input aperture was then defined in a process similar to the output aperture.

SUPPLEMENTARY NOTE 3: DEVICE CHARACTERIZATION PROCEDURE

The measurement setups used to characterize the spectrometer are schematically shown in Supplementary Figure **5**. Light from a tunable Ti-sapphire laser (SolsTiS, M-Squared) was coupled to a single mode optical fiber and collimated using a fiber collimator (F240FC-B, Thorlabs). A fiber polarization controller and a free space polarizer (LPVIS100-MP2, Thorlabs) were used to control the input light polarization, and different neutral density filters were used to control the light intensity. The beam illuminated the input aperture of the spectrometer at normal incidence. The focal plane of the spectrometer, located $\sim$200 μm away from the output aperture, was then imaged using a custom built microscope (objective: 100$\times$ UMPlanFl, NA=0.95, Olympus; tube lens: AC254-200-C-ML, Thorlabs; camera: CoolSNAP K4, Photometrics). Since the field of view is $\sim$136 μm (limited by the $\sim$15-mm image sensor, and the $\sim$111$\times$ magnification), while the total length over which the wavelengths are dispersed in the focal plane exceeds 1 mm, the objective is scanned along the dispersion direction to cover the whole focal plane at each wavelength (11 images captured for each wavelength). These images were then combined to form the full intensity distribution at each wavelength. The measurements were performed at 11 wavelengths (760 nm to 860 nm, 10-nm steps) to form the results shown in Figs. **4**b, **4**c , Supplementary Figure **6**, and

Supplementary Figure **7**. The measurements were also performed at a second set of wavelengths (761.25 nm, 811.25 nm, and 861.25 nm). These results are summarized in Fig. **4**d and Fig. **4**e for TE and TM polarizations. The resolution [Supplementary Figure **8**] was estimated by finding the full-width-half-maximum (FWHM) at each wavelength, and the displacement rate of the focus center along the y direction by changing the wavelength. The setup was slightly changed for measuring the focusing efficiencies. The input beam was partially focused by a lens (f =10 cm) such that all the beam power passed through the input aperture (with a $\sim$ 400 µm FWHM). In addition, the camera was replaced by a photodetector and a pinhole with a diameter of 3.5 mm in front of it to measure the focused power. The pinhole, corresponding to a $\sim$31-µm area in the focal plane, allows only for the in-focus light to contribute to the efficiency. The efficiency is then calculated at each wavelength by dividing these measured powers by the total power tightly focused by a 10-cm focal length lens that was imaged onto the power meter using the same microscope (i.e., by removing the spectrometer and the pinhole). The experimental setup for capturing the sample spectra is almost identical to Supplementary Figure **5**b, with the only difference of the polarizer being replaced by the sample of interest, and an 840-nm short-pass filter inserted before the sample. The light source was also replaced by a supercontinuum laser (Fianium Whitelase Micro, NKT Photonics).

SUPPLEMENTARY NOTE 4: ANGULAR RESPONSE MEASUREMENT

To measure the angular response of the device we used the setup shown in Supplementary Figure **9**c, equipped with a rotating stage with 0.1° precision in the x-z plane and 0.002° in the y-z plane. The collimator (connected to the fiber coming from the source) was mounted on this rotating stage, where the folded spectrometer was exactly located at its center. The incident angles were adjusted accordingly for 0°, ±0.3°, ±0.6°, ±1° angles. As can be observed in Supplementary Figure **9**a, the focal spots did not vary much in size as the angle is sweeped between -1° to +1° in x-direction. For measuring the tilt angle in the y-z plane, the distance from the collimator to the device was measured to be 280 mm. In order to impose ±0.15° tilt in y direction, the mounted collimator level is raised or lowered by 0.73 mm, and its tilt was adjusted accordingly such that the beam hits the center of the input aperture. As shown in Supplementary Figure **9**b, such a tilt in input incident angle does not degrade the spectral resolution of 1.25 nm.

SUPPLEMENTARY NOTE 5: INCREASED THROUGHPUT DESIGN

To further demonstrate the capabilities of the platform, we have designed a second spectrometer with significantly increased throughput. In order to achieve higher throughput, a larger input aperture was required, so the slab thickness was increased to 2 mm to give more freedom on the non-overlapping condition for the metasurfaces. The design, as shown in Supplementary Figure **10**, has a 2.5mm input aperture. To further improve the throughput, the acceptance angle of the device was increased. To achieve this goal, we took an approach similar to the fabricated spectrometer with the difference of adding extra phase terms to the input diffraction grating. This helps with orienting the focuses on the image plane for different incident angles, as well as relaxing the condition for focusing in the x-direction. This in turn allows for increasing the input incident angle to $\pm 15^{\circ}$ degrees. The phase profile coefficients for metasurfaces 1 to 3 in Supplementary Figure **10** are given in Supplementary Table **3**. In the final design, the power is distributed in an area close to 200 $\mu\mathrm{m}$ wide in the x-direction in the focal plane, instead of a diffraction limited focus. According to the intensity profiles shown in Supplementary Figure **10**b, the device can distinguish between wavelengths spaced by 0.5 nm both at the center wavelength of 810nm, and also at the side wavelengths of 760 nm and 860 nm. Based on the angular response of the device in the x-z and y-z planes, and also the input aperture size of the device, an etendue of around $\sim$13000 Sr$(\mu\mathrm{m})^2$ is estimated.

Supplementary Table 1. **Phase profile coefficients in terms of $[rad/mm^{m+n}]$ for metasurfaces 1 and 2**

Metasurface	$a_{x^2y^0}$	$a_{x^0y^2}$	$a_{x^2y^1}$	$a_{x^0y^3}$	$a_{x^4y^0}$	$a_{x^2y^2}$	$a_{x^0y^4}$	a_{ρ^6}	a_{ρ^8}	$a_{\rho^{10}}$
I (R=525.0 µm)	-4.02	-2.08	0.47	0.20	$-5.68e-4$	$7.55e-3$	$2.36e-3$	$1.93e-4$	$-3.22e-6$	$-5.886e-9$
II (R=582.5 µm)	-3.91	-3.70	-0.68	-0.24	$6.26e-3$	0.021	$6.34e-3$	$-2.48e-4$	$4.82e-6$	$-2.974e-9$

Supplementary Table 2. **Optimized grating post sizes [nm]** (D_x, D_y)

Optimization	D_{x1}	D_{y1}	D_{x2}	D_{y2}	D_{x3}	D_{y3}	D_{x4}	W_{y4}
Maximizing first order diffraction Efficinecy	93.4	93.4	117	117	132.8	132.8	155.4	155.4
Particle Swarm Optimization	68	134	115.2	119.6	147.4	151.2	137.8	178.8

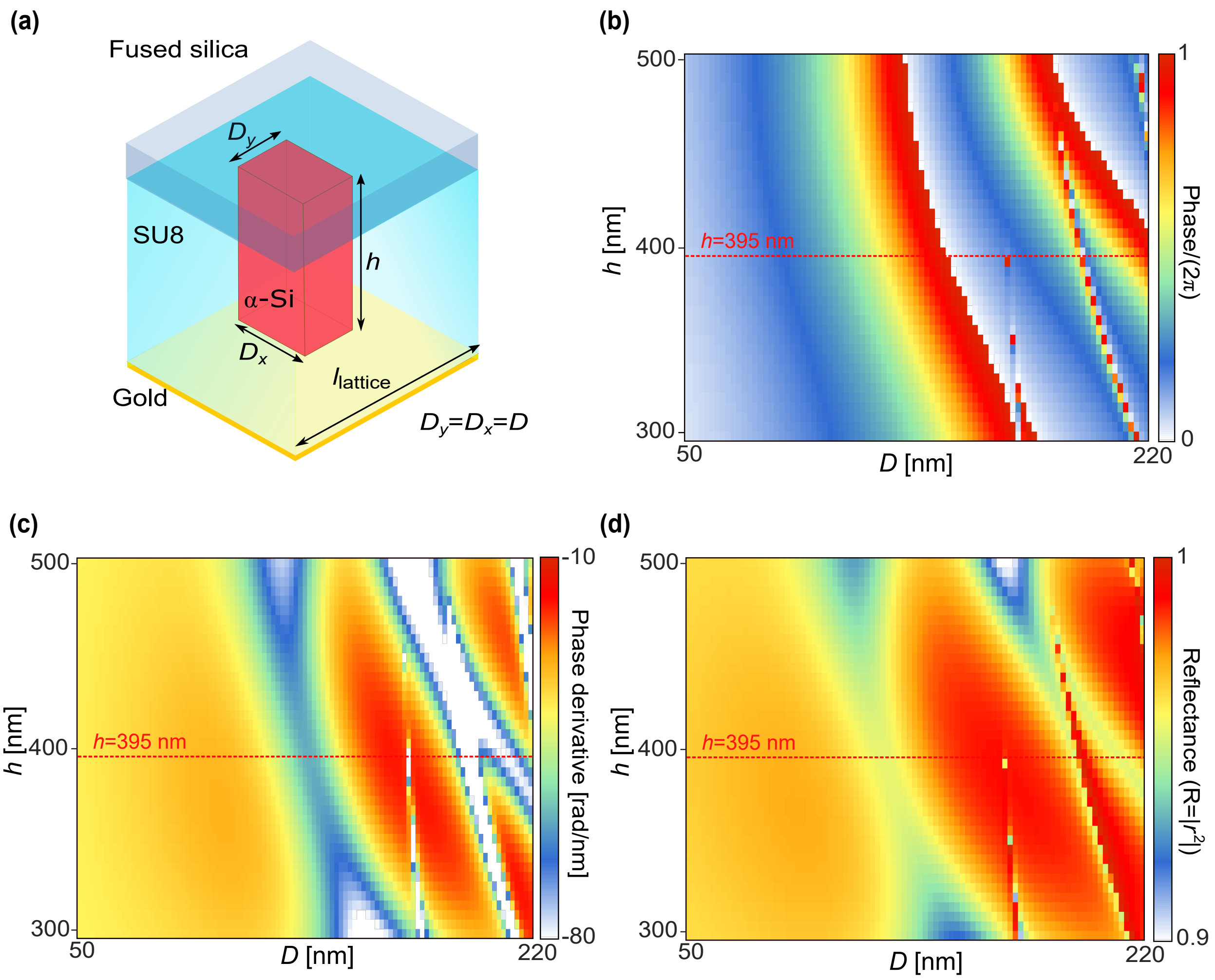

Supplementary Figure **1**. **Single-post periodic lattice simulations.** (**a**) Schematic of a rectangular post on top of a fused silica substrate, showing the post dimensions. The nano-posts are capped by a 2-µm-thick layer of SU-8, and backed by a reflective gold layer. (**b**) Simulated reflection phase under TE illumination with 33.9° incident angle. (**c**) Derivative of the phase with respect to the wavelength calculated and plotted versus the height (h) and width of the post (D_x=D_y=D). The nano-post height that provides full 2π phase coverage with high reflectance while minimizing variation of the phase derivative is found to be h=395 nm (the red line). (**d**) Reflectance as a function of post-width and height.

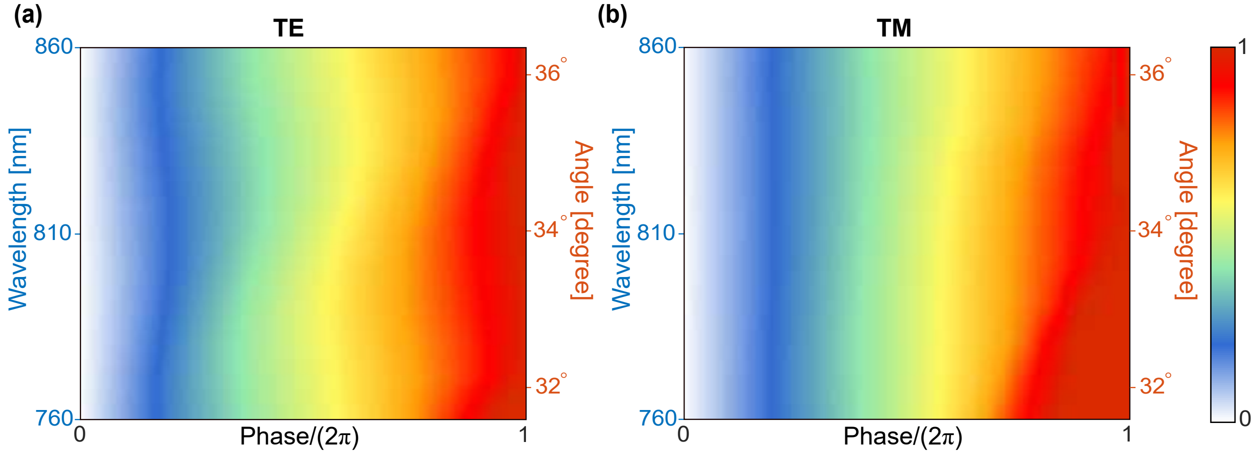

Supplementary Figure **2**. **Reflection phase variation versus wavelength.** (**a**) Reflection phase for TE polarized light from a uniform array of meta-atoms corresponding to the black curves in Fig. **3** versus wavelength. As depicted by the vertical axis on the right, the simulation for each wavelength is performed under an incident angle corresponding to the deflection angle of the input blazed grating at that wavelength. (**b**) Same as (**a**) for TM polarization.

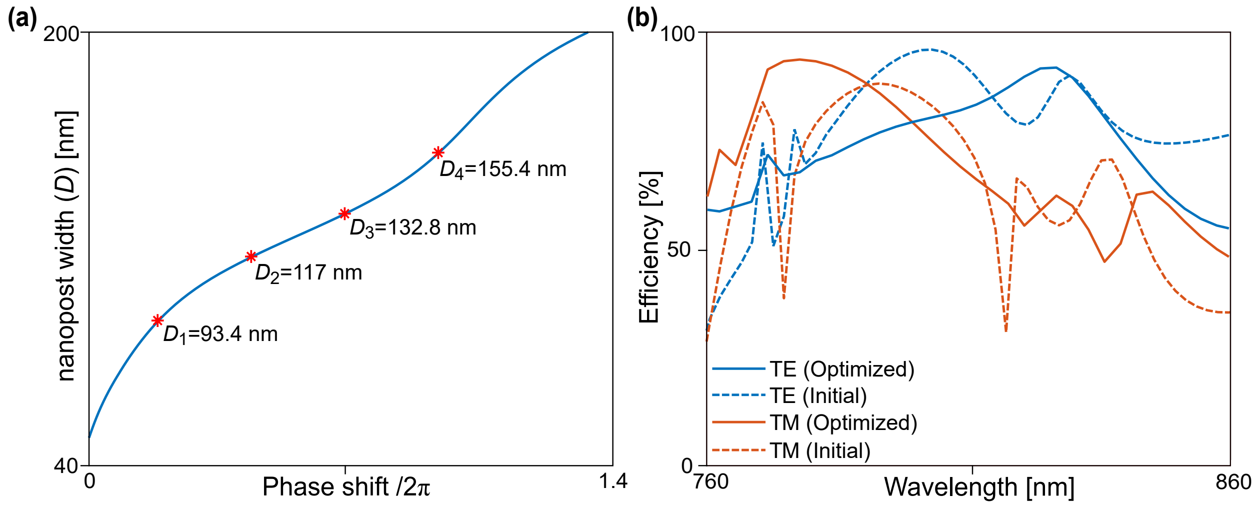

Supplementary Figure **3**. **Grating design curves and deflection efficiencies.** (**a**) Post width versus reflection phase for 395-nm-tall posts on a square lattice with a 250-nm lattice constant. The red stars correspond to the nano-post sizes found from this graph that have the highest deflection efficiency over the bandwidth. (**b**) TE and TM polarization deflection efficiency curves versus wavelengths for the initial (i.e., the red stars in (a)) and optimized nano-post dimensions given in Supplementary Table **2**.

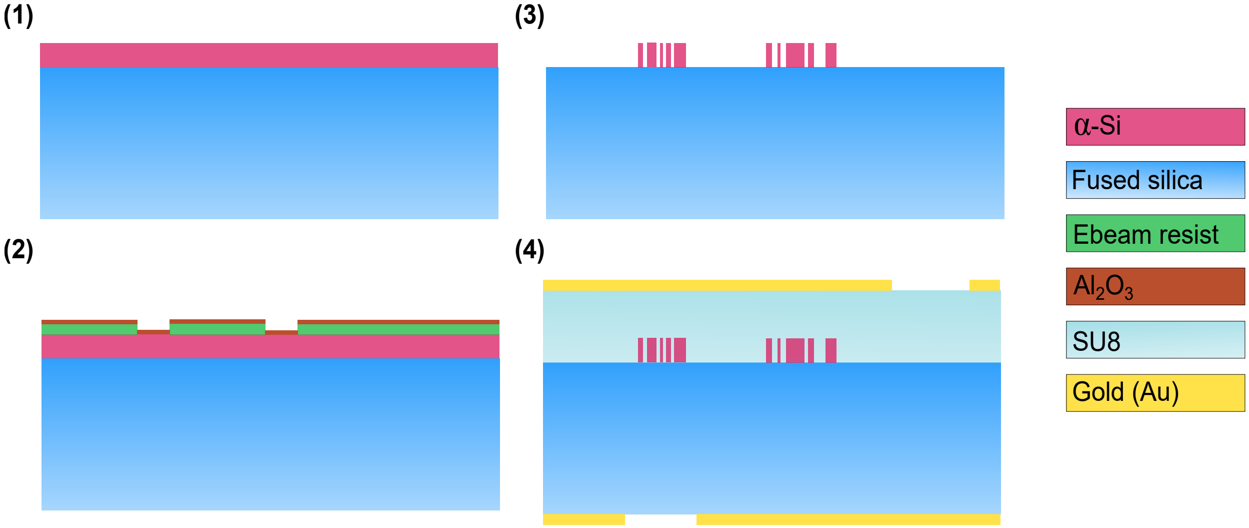

Supplementary Figure **4**. **The key fabrication steps.** A 395-nm-thick layer of α-Si is deposited on a 1-mm-thick fused silica substrate using PECVD. The metasurface pattern is generated with electron beam lithography, negated and transferred to the α-Si layer via lift-off and dry-etching processes. Both sides are covered with and SU-8 layer, and the input and output apertures are defined through photolithography and lift-off.

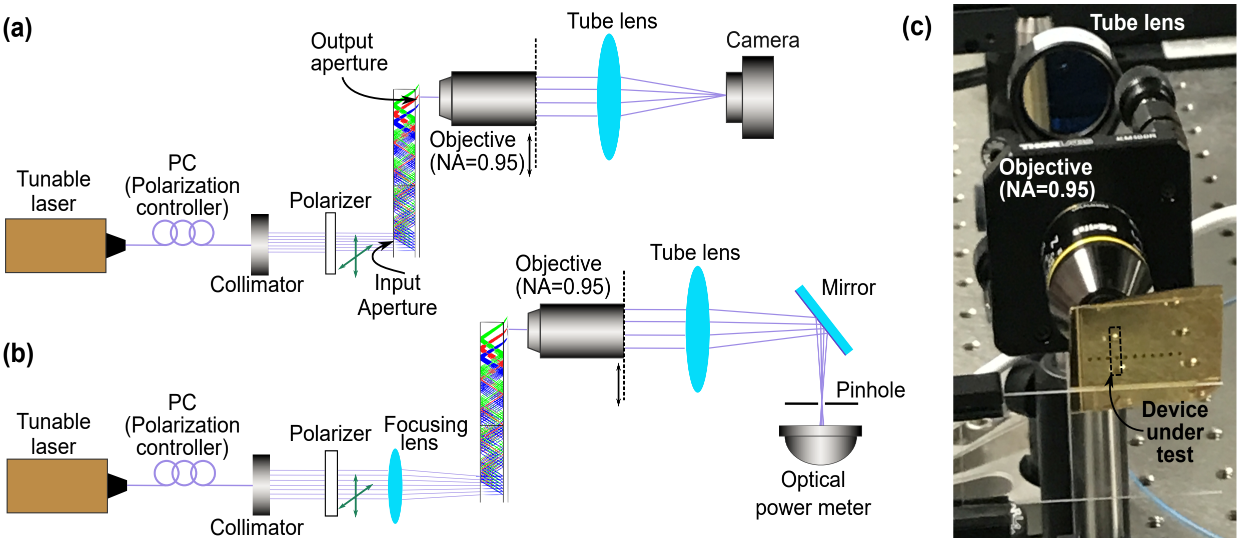

Supplementary Figure **5**. **Measurement setups.**(**a**) Schematics of the measurement setup used for device characterization. (**b**) Schematics of the setup used to measure the focusing efficiencies. (**c**) An optical image of a part of the actual measurement setup showing the device, the objective lens, and the tube lens.

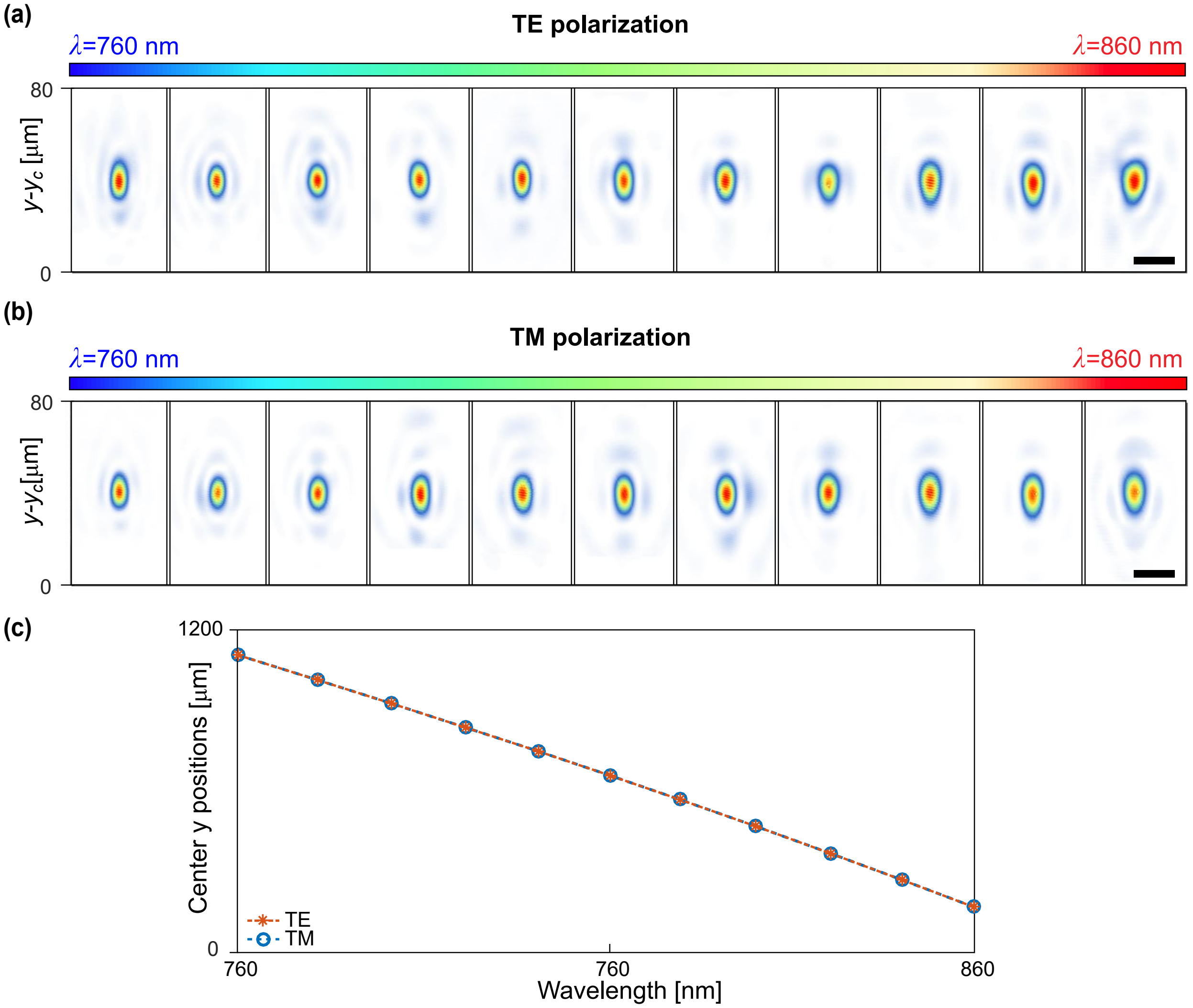

Supplementary Figure **6**. **Focal plane intensity profiles.** (**a**) Two-dimensional intensity profiles measured at several wavelengths (y_c is the center position of each profile) under illumination with TE polarized light, and (**b**) TM polarized light. (**c**) The position of the center of the focal spot along the dispersion direction, y, versus wavelength. The symbols represent the measured data, and the solid line is an eye guide. The scale bars are 20 μm.

Supplementary Table **3**. **Phase profile coefficients of the increased throughput design (Supplementary Figure 10) in terms of** $[rad/mm^{m+n}]$

Metasurface	$a_{x^2y^0}$	$a_{x^0y^2}$	$a_{x^2y^1}$	$a_{x^0y^3}$	$a_{x^4y^0}$	$a_{x^2y^2}$	$a_{x^0y^4}$	a_{ρ^6}	a_{ρ^8}	$a_{\rho^{10}}$
I (R=2.5 mm)	−0.44	−2.98	0.015	0.035	−1.5e−5	−6.16e−5	−3.1e−4	1.42e−7	−2.26e−10	−5.886e−13
II (R=1.5 mm)	−3.13	1.91	0.027	−0.04	−8.2e−4	−0.72e−4	3.8e−3	1.12e−5	−5.26e−8	7.58e−11
III (R=2.15 mm)	−2.45	−2.69	−0.067	−0.06	3.56e−4	−1.73e−5	−9.14e−4	6.71e−7	−1.72e−9	1.62e−12

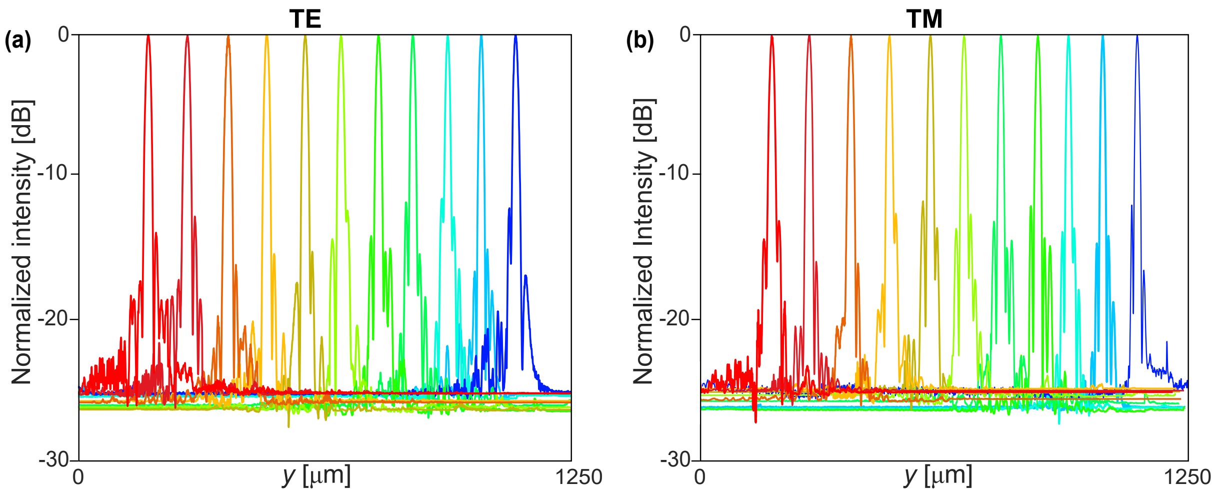

Supplementary Figure **7**. **Intensity distribution profiles on logarithmic scales** (**a**) Same information as Fig. **4**b and **4**c of the main text, plotted on logarithmic scale for TE polarization, and (**b**) for TM polarization.

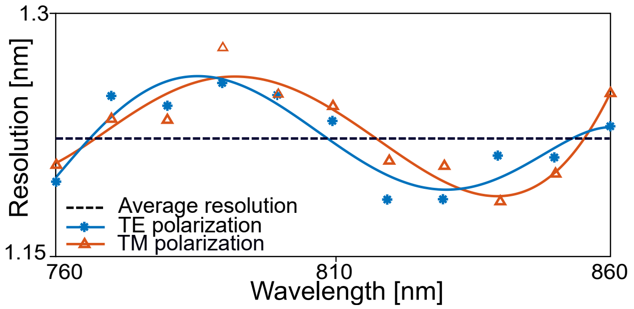

Supplementary Figure **8**. **Measured spectral resolution versus wavelength.** Spectral resolution estimated using the measured focal spot FWHM and the displacement rate of the focal spot with changing the wavelength. The average resolution is 1.22 nm for both polarizations. The symbols show the measured date and the solid lines are eye guides.

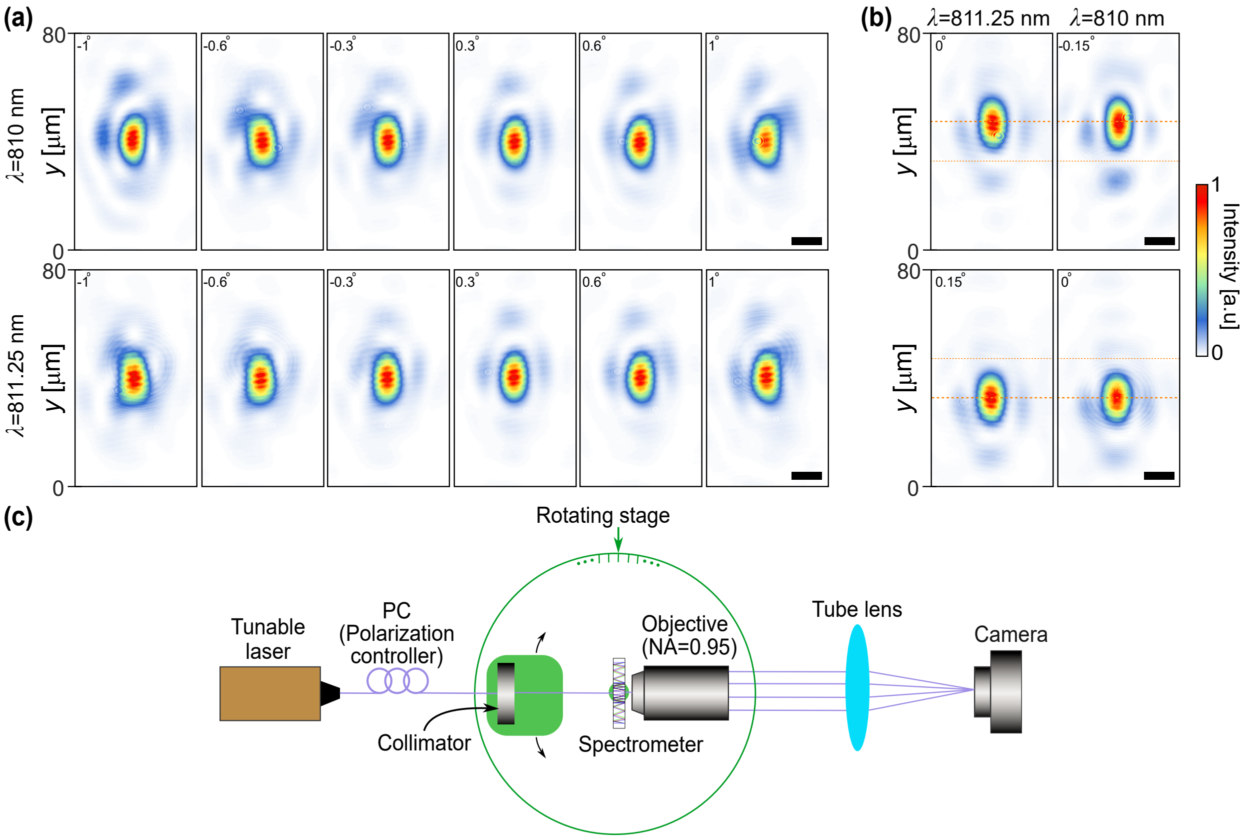

Supplementary Figure 9. **Measured angular response of the device for polar angle variation with respect to 0 angle in x-z and y-z planes.** (**a**) Angular response of the device for different tilted incident angles between -1° to +1° in the x-z plane. (**b**) Angular response of the device for ±0.15° tilted incident angles in the y-z plane. (**c**) experimental setup used for measuring the angular response. The scale bars are 10 μm.

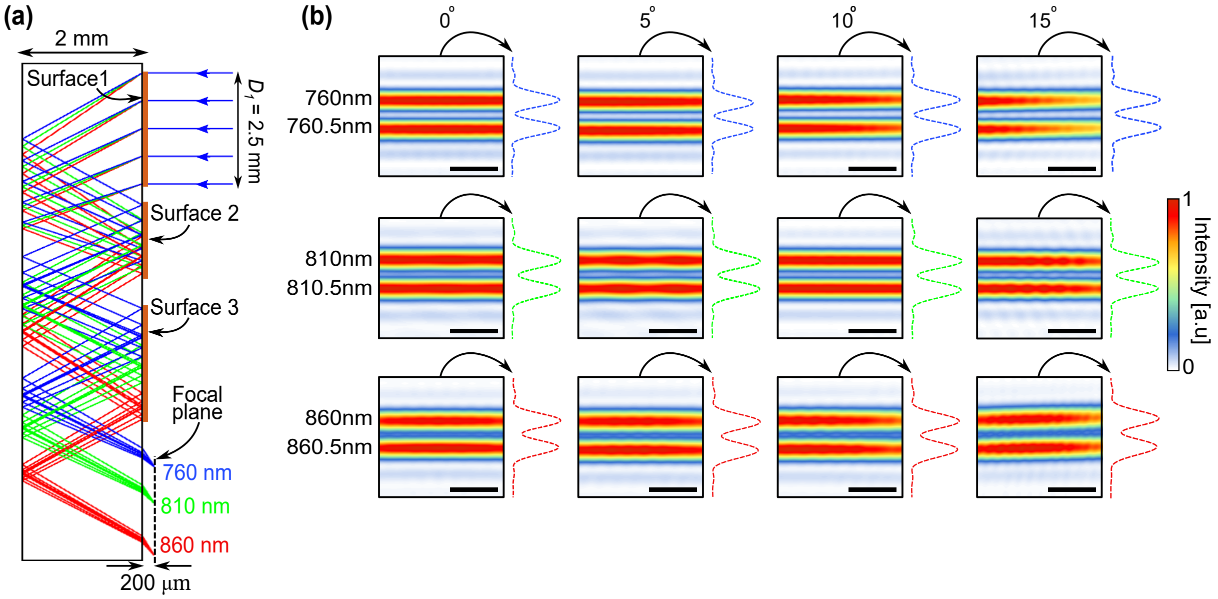

Supplementary Figure **10**. **Ray-optics design and simulation results of an extended-throughput folded spectrometer.** (**a**) Ray tracing simulation results of the extended-throughput folded spectrometer, shown at three wavelengths in the center and two ends of the band. The system consists of three metasurfaces optimized to separate different wavelengths of the light and focus them on the focal plane. (**b**) Simulated intensity distribution for two wavelengths separated by 0.5 nm around three different center wavelengths of 760 nm, 810 nm, and 860 nm for 4 different incident angles of 0°, 5°, 10° and 15°. The intensity distributions show that wavelengths separated by 0.5 nm are theoretically resolvable for all aforementioned incident angles. The scale bars are 15 µm.

SUPPLEMENTARY REFERENCES

[1] Liu, V. & Fan, S. S 4: A free electromagnetic solver for layered periodic structures. *Computer Physics Communications* **183**, 2233–2244 (2012).

[2] Kamali, S. M., Arbabi, E., Arbabi, A., Horie, Y. & Faraon, A. Highly tunable elastic dielectric metasurface lenses. *Laser & Photonics Reviews* **10**, 1002–1008 (2016).

[3] Arbabi, A. *et al.* Increasing efficiency of high-na metasurface lenses (conference presentation). In *SPIE OPTO*, 101130K–101130K (International Society for Optics and Photonics, 2017).
