# Supplementary material for: Compact folded metasurface spectrometer
Source: Nat Commun. 2018 Oct 10;9:4196. doi: 10.1038/s41467-018-06495-5 (PMC6180047; doi:10.1038/s41467-018-06495-5)
Supplement: Supplementary file 2 — Peer Review File [file 41467_2018_6495_MOESM2_ESM.pdf]

**Editorial Note:** This manuscript has been previously reviewed at another journal that is not operating a transparent peer review scheme. The manuscript was considered suitable for publication without further review at Nature Communications.
